# Isolation, identification, and significance of salivary *Veillonella* spp., *Prevotella* spp., and *Prevotella salivae* in patients with inflammatory bowel disease

**DOI:** 10.3389/fcimb.2023.1278582

**Published:** 2023-11-20

**Authors:** Moshira I. Hammad, Georg Conrads, Mohamed M. H. Abdelbary

**Affiliations:** ^1^ Division of Oral Microbiology and Immunology, Department of Operative Dentistry, Periodontology and Preventive Dentistry, Rheinisch-Westfälische Technische Hochschule University Hospital, Aachen, Germany; ^2^ Division of Nosocomial Pathogens and Antibiotic Resistances, Department of Infectious Diseases, Robert Koch Institute, Wernigerode, Germany

**Keywords:** *Veillonella* spp., *Prevotella* spp., *Prevotella salivae*, inflammatory bowel disease, real-time quantitative PCR, 16S rRNA amplicon sequencing

## Abstract

The global prevalence of inflammatory bowel disease (IBD) is on the rise, prompting significant attention from researchers worldwide. IBD entails chronic inflammatory disorders of the intestinal tract, characterized by alternating flares and remissions. Through high-throughput sequencing, numerous studies have unveiled a potential microbial signature for IBD patients showing intestinal enrichment of oral-associated bacteria. Simultaneously, the oral microbiome can be perturbed by intestinal inflammation. Our prior investigation, based on 16S rRNA amplicon sequencing, underscored elevated abundance of *Veillonella* spp. and *Prevotella* spp. in the salivary microbiomes of IBD patients. Noteworthy, *Prevotella salivae* emerged as a distinct species significantly associated with IBD. *P. salivae* is an under-recognized pathogen that was found to play a role in both oral and systemic diseases. In this study, we delve deeper into the salivary microbiomes of both IBD patients and healthy controls. Employing diverse cultivation techniques and real-time quantitative polymerase chain reactions (RT-qPCR), we gauged the prevalence and abundance of *Veillonella* spp., *Prevotella* spp., and *P. salivae*. Our isolation efforts yielded 407 and 168 strains of *Veillonella* spp., as well as 173 and 90 strains of *Prevotella* spp., from the saliva samples of IBD patients and healthy controls, respectively. Veillonella-vancomycin agar emerged as the discerning choice for optimal *Veillonella* spp. cultivation, while Schaedler kanamycin-vancomycin agar proved to be the most suitable medium for cultivating *Prevotella* spp. strains. Comparing our RT-qPCR findings to the previous 16S rRNA amplicon sequencing data, the results corroborated the higher abundance of *Veillonella* spp., *Prevotella* spp., and *P. salivae* in the saliva of IBD patients compared to healthy controls. However, it’s worth noting that in contrast to RT-qPCR, the 16S rRNA amplicon sequencing data revealed greater absolute abundance of all three bacterial groups in both IBD patients and controls.

## Introduction

A healthy individual produces and swallows about 1.5 liters of saliva each day, harboring a huge number of oral colonizing bacteria (Edgar, 1992; Humphrey and Williamson, 2001; Nasidze et al., 2009). The bacterial microbiota inhabiting the oral cavity and the gut are widely diverse (Donaldson et al., 2016; Lloyd-Price et al., 2017; Mark Welch et al., 2019). The body’s mucosal surfaces, including the gut and oral mucosa, are colonized by a complex ecosystem (Chattopadhyay et al., 2019). Given the concept that the gut mucosa can be viewed as a continuum of the oral mucosa, thereby bridging the gap between the mouth and the gastrointestinal tract, it is conceivable that the oral cavity might serve as a reservoir for both intestinal commensals and potential pathogens (Braga and Squier, 1980; Bartlett et al., 2020; Balto et al., 2023). It was previously suggested that swallowed oral microbes may disrupt the homeostasis of the gut microbiota leading to dysbiosis (Donoff et al., 2014; Elad et al., 2019; Bao et al., 2022). This dysbiosis could bi-directionally affect other body organs leading to chronic diseases that affect the cardiac, pulmonary, and nervous systems (Nicholson et al., 2012; Forkosh and Ilan, 2019; Byrd and Gulati, 2021). The exact interaction mechanism between the gut and other organs is still elusive. In addition, oral and gut microbiota could play a role either as commensals or as harmful agents, which is directed and controlled by the host defense mechanisms (Lamont and Hajishengallis, 2015). Due to its widespread prevalence and frequent occurrence, inflammatory bowel disease (IBD) has drawn the attention of various researchers. IBD is a set of chronic intestinal inflammatory disorders marked by repeated stages of remission and relapse. IBD can be divided into two main clinical groups: Crohn’s disease (CD) and ulcerative colitis (UC). It is considered to be a multifactorial disease in which genetics, the immune response, diet, medications, bad oral hygiene, and smoking play etiological roles (de Souza and Fiocchi, 2016). For over a decade, it has been asserted that UC has a psychosomatic origin. Numerous studies have shown that UC patients have a greater incidence of psychological disorders than healthy controls (Andrews et al., 1987; Tarter et al., 1988; Magni et al., 1991). Others, however, debated that stress may contribute to the development and exacerbations of the disease (Greene et al., 1994; Mawdsley and Rampton, 2005). Till now the exact etiology of IBD is not fully discovered. From a microbiological perspective, individuals with IBD exhibit dysbiosis within their oral and gut microbiota, a condition believed to significantly exacerbate the disease’s progression. IBD does not originate from a solitary causative microorganism; instead, the complete microbiome and its corresponding immunological response jointly assume a pivotal role in its etiology (Aldars-García et al., 2021). Furthermore, IBD is intricately linked to compromised diversity in both oral and gut microbial ecosystems, resulting in fluctuations in the abundance of specific taxa (Manichanh et al., 2006; Brennan and Garrett, 2019; Olsen and Yamazaki, 2019; Pittayanon et al., 2020). In a recent study, we demonstrated that IBD patients’ saliva contained more *Veillonella* spp. and *Prevotella* spp. bacteria than that of healthy controls (Abdelbary et al., 2022). This is in accordance with other studies revealing that *Prevotella* spp. was more prevalent in active CD compared to the remission phase (Zhang et al., 2020). Said et al. found that the microbial richness and diversity did not change in IBD patients, rather, the composition of the salivary microbiota differed from that of healthy individuals. This was demonstrated by the significantly higher levels of salivary *Veillonella* spp. and *Prevotella* spp. in both UC and CD individuals compared to healthy controls (Said et al., 2014). *Veillonella* spp. and *Prevotella* spp. bacteria are commonly encountered as commensals in the oral cavity, yet they can also serve as causative agents (pathobionts) of periodontal and endodontic infections (Rôças and Siqueira, 2009; Hsiao et al., 2012; Rajaram et al., 2016; Shin et al., 2018). Prolonged imbalances within the mucosa-attached oral microbiota may potentially give rise to systemic diseases, particularly as pathobionts gain access to the intestine via the bloodstream (Georges et al., 2022). Alternatively, the ingestion of microbes linked to periodontitis could disrupt the delicate equilibrium of the gut microbiota (Donoff et al., 2014; Elad et al., 2019; Bao et al., 2022). In contrast to non-IBD controls, the prevalence of periodontitis is notably elevated among CD patients. (Van Dyke et al., 1986; Brandtzaeg, 2001; Brito et al., 2008; Vavricka et al., 2013). This shows that the oral-gut axis is essentially an uninterrupted pipeline linking the oral cavity to the gut. In our preceding study, (Abdelbary et al., 2022), a noteworthy discovery emerged as *Prevotella salivae* exhibited a significant link to IBD patients, with its operational taxonomic unit (OTU14) being remarkably raised in their saliva samples. *P. salivae*, despite being an often overlooked pathogen, has demonstrated its involvement in various conditions, including periodontitis (Sakamoto et al., 2004), infections within root canals, periapical abscesses (Hsiao et al., 2012), and caries (Jiang et al., 2016). Moreover, case reports have further linked *P. salivae* to peritonitis (Domingues et al., 2023) and bacteremia (Avanzato et al., 2023).

In light of these findings, the present study amalgamated traditional cultivation techniques with molecular-based methodologies to ascertain both the prevalence and significance of *Veillonella* spp., *Prevotella* spp., and *P. salivae* in the saliva of IBD patients in comparison to healthy controls. Additionally, we devised a novel RT-qPCR assay for the detection of *P. salivae*. Furthermore, we conducted a comparative analysis of the absolute abundance levels derived from the RT-qPCR assays for the three groups—*Veillonella* spp., *Prevotella* spp., and *P. salivae*—with the 16S rRNA amplicon sequencing data derived from the prior study (Abdelbary et al., 2022).

## Materials and methods

### Study design and sample collection

In our previous study (Abdelbary et al., 2022), a cohort of 14 IBD patients and 12 healthy controls was assembled within the timeframe spanning from March 2019 to April 2021 at the RWTH Aachen University Hospital. Clinical data including gender, age, disease type, and exclusion criteria for patients and healthy controls were documented in that study (Abdelbary et al., 2022), which was approved by the Ethics Committee of the RWTH Aachen University Hospital (No. EK 069/19 for IBD patients and No. EK206/09 for healthy control subjects) and conducted in accordance with the Declaration of Helsinki. All participants signed a written informed consent before sample donation. Saliva samples were diligently collected from both patients and healthy controls using sterile 100 ml containers (Sarstedt, Nümbrecht, Germany). Patient samples were denoted by the letter “P” followed by the patient number, while healthy control samples were indicated with the letter “C” followed by the corresponding number. Sampling occurred in the morning, with participants instructed to chew paraffin gum to stimulate saliva production before collecting their samples. There were no dietary restrictions imposed on the participants; however, all individuals were kindly asked to abstain from eating, drinking, or brushing their teeth for a minimum of approximately 30 minutes before the sampling process. Following collection, each saliva sample was meticulously divided into aliquots and subsequently stored at a temperature of -72°C.

### Cultivation and isolation of *Veillonella* spp., *Prevotella* spp., and *Prevotella salivae*

In the context of identifying the presence of *Veillonella* spp., *Prevotella* spp., and *P. salivae* in saliva samples, a methodical selection of agar media was undertaken, taking into consideration their distinct growth requirements and characteristics. It is pertinent to mention that various species within these genera may demonstrate differing growth patterns on various media. Hence, we meticulously assessed the efficacy of diverse agar media in isolating *Veillonella* spp., *Prevotella* spp. and *P. salivae*. This evaluation was carried out using conventional cultivation and isolation methods. The agar media utilized for this purpose comprised: 1) Veillonella agar base +/- vancomycin M416 (HiMedia Laboratories, GmbH, Einhausen, Germany), previously utilized for the selective isolation of *Veillonella* species (Nakhaee et al., 2018; Stojanovski et al., 2021), 2) Blood Agar (Tryptic Soy Agar with sheep blood; Oxoid, Wesel, Germany), a nutrient rich, non-selective medium renowned for isolating and cultivating a wide range of both fastidious and non-fastidious microorganisms (Potempa et al., 2009; Ono et al., 2022), 3) Brucella Agar (BD Biosciences, Heidelberg, Germany), employed for the isolation and cultivation of diverse fastidious and non-fastidious microorganisms including different oral bacterial species such as *Prevotella* spp. (Jousimies-Somer, 1995; Tyrrell et al., 2003), 4) GC–Lect (BD Biosciences, Heidelberg, Germany), was previously designed for the isolation and cultivation of fastidious bacteria (Gatti et al., 2010), 5) non-selective Schaedler agar (BD Biosciences, Heidelberg, Germany), used for the isolation and cultivation of anaerobic microorganisms including oral bacteria (Ileri Keceli et al., 2015; Kau et al., 2021), and 6) Schaedler agar enriched with kanamycin/vancomycin (KV; BD Biosciences, Heidelberg, Germany) agar plates, intended for the isolation and cultivation of fastidious gram-negative anaerobic microorganisms such as *Veillonella* spp., *Prevotella* spp. and *P. salivae* (Banche et al., 2007; Cieplik et al., 2020; Kau et al., 2021). To achieve this objective, we employed sterile 10 μl inoculation loops to evenly distribute the samples, facilitating the growth of individual colonies. Subsequently, the agar plates were placed in an incubator set at 37°C, creating an anaerobic environment maintained through the use of anaerobe pouch system sachets, coupled with an indicator (GasPak™ EZ, BD, Heidelberg, Germany). Typically, a period of 3 to 5 days proved optimal for ensuring robust growth. In certain instances, extended durations of up to one week or more were requisite to achieve satisfactory growth. For further isolation, additional sub-culturing was performed on agar plates, with this process repeated three to four times to yield the desired isolates. Notably, *Prevotella* spp. isolates were subjected to ultraviolet (UV) light visualization, a technique employed to identify distinct colonies exhibiting a characteristic brick-red fluorescence, setting them apart from other pigmented genera. The isolated strains underwent identification using the Matrix-assisted LASER Desorption/Ionization-Time of Flight device (MALDI-TOF MS Biotyper, Bruker Daltonik GmbH, Bremen, Germany). Subsequently, all saliva samples were subjected to dilution with Brain Heart Infusion (BHI) broth, maintaining a ratio of 1:4. To further enhance the proliferation of Gram-negative anaerobic *P. salivae* bacteria, while restraining the growth of Gram-positive counterparts, the diluted saliva samples were once again streaked onto Schaedler KV agar plates and incubated at 37°C under anaerobic conditions.

### Growth and inhibition zones from bacterial cross streaks

During the endeavor to isolate *P. salivae*, a distinctive reliance on the presence of *Prevotella melaninogenica* came to light, prompting the exploration of co-culture techniques. Utilizing Schaedler KV agar plates, five discrete strains of *P. melaninogenica* (OMI1181, OMI1288, OMI1328, OMI1431, and OMI1432) were concurrently streaked. Subsequent to this, six strains of *P. salivae* (including the reference strain DSMZ 15606, as well as OMI1311, OMI1349, OMI1399, OMI1400, and OMI1430) were horizontally streaked in six parallel lines perpendicular to the streaks of *P. melaninogenica*. Importantly, all chosen *P. salivae* strains had been isolated from the very same samples as the *P. melaninogenica* strains.

These plates were maintained within an anaerobic environment at 37°C, and they underwent three separate checks over a span of 2 to 7 days. The growth of both *P. melaninogenica* and *P. salivae* was meticulously monitored, with particular focus on the intersections of the vertical and horizontal streaks. Furthermore, a pairwise and triplet arrangement of the same strains was replicated on separate Schaedler KV agar plates. Following the attainment of substantial growth, the utilization of MALDI-TOF assisted in the meticulous analysis of colony identity, especially in cases of uncertainty, such as those arising in the intersection regions.

### DNA extraction, amplification and sequencing of 16S rRNA gene

Among the collected samples from both IBD patients and healthy controls, a total of 41 *Veillonella* spp. strains and 28 *Prevotella* spp. strains, which had been successfully isolated multiple times, were chosen for further evaluation. Specifically, a single representative isolate for each species was selected from each patient or healthy control. The biomass of each isolated strain was carefully gathered using sterile 10 μl inoculation loops and subsequently re-suspended within 1.5 ml Eppendorf tubes, with each tube containing 1 ml of 0.9% sodium chloride solution. The tubes were centrifuged at 8,000 rpm for 1 minute and the supernatant was discarded. For molecular based identification, DNA extraction was performed from all isolated *Veillonella* spp.*, Prevotella* spp., and *P. salivae* strains using the QIAamp^®^ DNA Mini Kit (Qiagen, Hilden, Germany) according to the manufacturer’s instructions. DNA was then frozen and stored at -72°C. Subsequently, amplification of the 16S rRNA gene (V1-V9) was performed using the universal forward primer pF1: 5’ AGAGTTTGATCCTGGCTCAG and the universal reverse primer pR1: 5’ GGCTACCTTGTTACGACTT (Conrads et al., 1997). PCR was performed under the following conditions: an initial denaturation step at 94°C for 2 minutes, followed by 35 cycles of: denaturation at 94°C for 30 seconds, annealing at 50°C for 30 seconds, and elongation at 72°C for 90 seconds. The PCR was terminated by a final extension step at 72°C for 10 minutes. PCRs included a negative control and positive control. PCR product purification was then performed using the NucleoSpin gDNA kit (Machery-Nagel, 2012/Rev.02, Dueren, Germany) according to the manufacturer’s instructions. Sanger sequencing was performed bi-directionally using both forward and reverse primers. Sequence contigs were also generated bi-directionally using both forward and reverse sequences. The gene sequences were analyzed using the Basic Local Alignment Search Tool (BLAST) to accurately identify the species of all the investigated isolates (Altschul et al., 1990). Chromatograms were compared to reference strains to reveal and correct ambiguities (**Table S3**). A multi-fasta file of all gene sequences was aligned using the ClustalW algorithm implemented in the MEGA software version 11 (Tamura et al., 2021). Subsequently, the multiple sequence alignments were used to construct a neighbor-joining phylogenetic tree using the default settings and the 1000 replicates bootstrap in MEGA11 for both *Veillonella* spp. and *Prevotella* spp. strains.

### Primer design and quantitative PCR

RT-qPCR method was performed to quantify and further specify sequences of the three groups: *Veillonella* spp., *Prevotella* spp., and *P. salivae.* The 331F/797R primers were chosen for the quantification of total bacteria, as the primers target the V3-V4 regions (Jian et al., 2020). The Veil F/R primers (Rinttilä et al., 2004) were chosen for quantification of genus *Veillonella*. The g-Prevo F/R primers (Matsuki et al., 2002) were used for quantification of the genus *Prevotella*. Subsequently, the P.s F/R primers were designed for quantification of *P. salivae* based on the 16S rRNA gene (accession number AB108826) and OTU14 sequences which were detected in the previous study (Abdelbary et al., 2022). The primers were designed and in-silico tested using Primer-BLAST tool. A list of primers used in this study and their coverage is summarized in **Table 1**. Total bacteria, *Veillonella* spp., *Prevotella* spp., and *P. salivae* were quantified using 1 ul of saliva DNA. Briefly, the thermal cycling conditions were as follows: 1) denaturation at 95° C for 15 seconds, followed by 40 cycles for P.s primers and 35 cycles for all other primers (see **Table 1**), 2) annealing at a primer-specific temperature (see **Table 1**) for 15 seconds, 3) extension at 72°C for 1 minute, and 4) melting curve stage of 95°C for 15 seconds, 60°C for 1 minute and 95°C for 15 seconds. The 10-log-fold standard curves ranged from 10^1^ to 10^8^. Quantitative estimate of the initial template concentration in a sample was calculated by linear extrapolation of data from standard curves. All RT-qPCR assessments were performed in triplicates for each primer set and sample. PCR steps included negative controls to screen for contamination by exogenous DNA.

**Table 1:** List of primers used in this study and their coverage.

Target	Primer sequence (5’-3’)	Amplicon size in base pair	Annealing	Reference organism	Coverage (%)*	#PCR cycles	References
*P. salivae*	P.s F: 5’- ACCTTATgAggTTTTCAgCAgAC	527	54°C	*P. salivae*	100**	40	
	P.s R: 5’- CTAAgCATTTCACCgCTACACg						
*Prevotella* spp.	g-Prevo F: 5’ - CACRgTAAACgATggATgCC	527-529	52°C	*P. salivae*	65.8	35	Matsuki et al., 2002
	g-Prevo R: 5’ - ggTCgggTTgCAgACC						
*Veillonella* spp.	Veil F: 5’ - AYCAACCTgCCCTTCAgA	343	58°C	*V. parvula*	72	35	Rinttilä et al., 2004
	Veil R: 5’ - CgTCCCgATTAACAgAgCTT						
Bacteria qPCR	331F: 5’ - TCCTACgggAggCAgCAgT	466	57°C	*S. mutans*	Total bacteria 68.4	35	Jian et al., 2020
	797R: 5’- ggACTACCAgggTATCTAATCCTgTT				*Prevotella 98.4*		
					*Veillonella 0.2*		
16S rRNA gene (V1-V9)	pF1: 5’ - AgAgTTTgATCCTggCTCAg	1490-1524	50°C		Total bacteria 26.1	35	Conrads et al., 1997
	pR1: 5’ - ggCTACCTTgTTACgACTT				*Prevotella* 25.8		
					*Veillonella* 33.5		
16S rRNA amplicon sequencing (V3-V4)	341F: 5’ CCTACGGGNGGCWGCAG	444	55°C	*S. mutans*	Total bacteria 82.8	35	Abdelbary et al., 2022
	785R: 5’ GACTACHVGGGTATCTAATCC				*Prevotella* 90.4		
					*Veillonella* 81.5		

*The coverage (i.e. the percentage of potentially covered taxa within the target) was estimated using the SILVA TestPrime tool, allowing no mismatches during primer annealing.

**According to primer BLAST results from 07/08/2023.

### Statistical analysis and calculation of absolute abundance

Absolute abundance analysis was conducted on the three specified groups: *Veillonella* spp., *Prevotella* spp., and *P. salivae*, encompassing both IBD patients and healthy controls. This analytical approach was applied to both datasets, namely the RT-qPCR and the 16S rRNA amplicon sequencing data, with the latter sourced from the previous study (Abdelbary et al., 2022).

In the context of RT-qPCR, the absolute abundance values were directly derived from the PCRs executed with the three specific primers, as elucidated earlier. Conversely, for the 16S rRNA amplicon sequencing, the determination of absolute abundance was executed in accordance with the methodology outlined in a prior study (Jian et al., 2020). This entailed multiplying the relative abundance of each taxon by the overall bacterial count. The quantification of the total bacterial count was performed in this study through RT-qPCR, employing the specific primer set 331F/797R as previously mentioned. Statistical analysis was conducted through the utilization of the Kruskal-Wallis test, with GraphPad Prism software version 9.4.1 (LLC., San Diego, CA, USA) serving as the analytical tool.

## Results

### High prevalence of *Veillonella* spp. and *Prevotella* spp. in IBD

Instead of only focusing on DNA sequences, which might not necessarily reflect the presence of viable bacterial cells, it holds significance to encompass the monitoring of viable bacterial cell existence through the process of cultivating and identifying bacterial strains at the species level. To this effect, our approach led to the isolation of a total of 407 *Veillonella* spp. strains from the saliva samples of IBD patients, in contrast to 168 strains isolated from healthy controls. In a parallel manner, 173 strains of *Prevotella* spp. were successfully isolated from the same samples for IBD patients, and 90 strains for healthy controls.

We did not detect any *Veillonella* spp. strains in patients P3 and P6. Nor did we detect any *Prevotella* spp. strains in patients P3, P10, P12, P13, and P21. Remarkably, a substantial portion of 152 *Veillonella* spp. strains were effectively isolated using Veillonella/vancomycin agar, thus establishing it as the preferred selective medium for *Veillonella* spp. bacteria. Moreover, 104 *Veillonella* spp. strains were isolated from Brucella agar, and an additional 98 strains were obtained from Schaedler agar media. Among the *Veillonella* species isolated, *V. dispar* emerged as the most predominant in IBD patients, constituting 48.6% of the *Veillonella* species, followed by *V. parvula*, accounting for 33%. *atypica* exhibited a comparatively lower prevalence, representing only 17% of the *Veillonella* species in IBD patients (**Table S1**). In the context of healthy controls, *Veillonella* spp. strains were effectively isolated using Veillonella/vancomycin agar, resulting in the isolation of 98 strains. However, the number of isolated strains decreased when using Brucella, Blood agar, and Schaedler agar media, yielding 27, 19, and 14 strains, respectively (**Table 2**). In contrast, the growth of *Veillonella* spp. bacteria exhibited poor results on Schaedler KV agar or GC-Lect agar media. Notably, the most prevalent *Veillonella* species among healthy controls was *V. parvula*, accounting for 57% of the total isolated *Veillonella* species. Following this, *V. dispar* constituted 37%, with *V. atypica* representing a smaller proportion at 7.7% (**Table S1**). Generally, Blood agar, Schaedler-KV agar, and GC-Lect agar displayed limited effectiveness in promoting the growth of *Veillonella* spp. bacteria (**Table 2**).

**Table 2:** Total and average number of isolated Veillonella spp. and Prevotella spp. strains in IBD patients and healthy controls.

Agar type	Total no. of isolated *Veillonella* spp.		Average of isolated *Veillonella* spp.		Total no. of isolated *Prevotella* spp.		Average of isolated *Prevotella* spp.	
	patients	controls	per patient	per control	patients	controls	per patient	per control
**Blood**	37	19	2.5	1.6	11	1	0.7	0.1
**Brucella**	104	27	6.9	2.3	10	5	0.6	0.4
**GC-Lect**	0	1	0	0.1	6	7	0.4	0.6
**Veil/vancomycin**	152	98	10.1	8.2	63	3	4.2	0.3
**Schaedler**	98	14	6.5	1.2	5	0	0.3	0
**Schaedler KV**	16	9	1.1	0.7	78	74	5.2	6.2
**Total isolates**	**407**	**168**			**173**	**90**		

Transitioning to the isolation of *Prevotella* spp. from IBD patients, Schaedler KV agar emerged as the most suitable medium. Our findings indicated that the growth of *Prevotella* spp. bacteria was not significantly enhanced by Blood, Brucella, GC-Lect, and non-selective Schaedler agar media. These media resulted in the isolation of only 11, 10, 6, and 5 *Prevotella* spp. strains, respectively. In IBD patients, *P. melaninogenica* constituted 45.6% of the isolated *Prevotella* species, followed by *P. histicola* at 32.9%. Additionally, *P. veroralis*, *P. nanceiensis*, and *P. maculosa* made up 11.56%, 5.2% and 0.4% respectively (**Table S2**). Likewise, for healthy controls, Schaedler KV medium proved to be the prime medium for *Prevotella* spp., yielding identification of 74 strains. Once again, *P. melaninogenica* emerged as the most predominant *Prevotella* species, accounting for 63% of the composition. *P. nanceiensis*, *P. histicola*, and *P. loescheii* comprised smaller proportions of 12%, 6.6% and 3.3% respectively (**Table S2**). Despite the inherent non-quantitative nature of isolation procedures, the discernible contrast in *P. histicola* prevalence was striking.

### Isolation and identification of *P. salivae*

It’s important to note that despite the frequent detection of *P. salivae* through molecular techniques like 16S rRNA amplicon sequencing (OTU14) (Abdelbary et al., 2022), its isolation posed a significant challenge, requiring multiple attempts to successfully cultivate a limited number of strains. Initially, a single isolate of *P. salivae* was found in control C3 on selective Schaedler KV agar plates prior to any dilution. In a subsequent effort, dilution of saliva samples with BHI broth provided a slight facilitation of isolation. In this instance, successful isolation of *P. salivae* was achieved from the saliva samples of P1, P16, C3, C5, and an additional freshly collected healthy control sample (C17), all applied on Schaedler KV agar plates.

**Figure 1:**
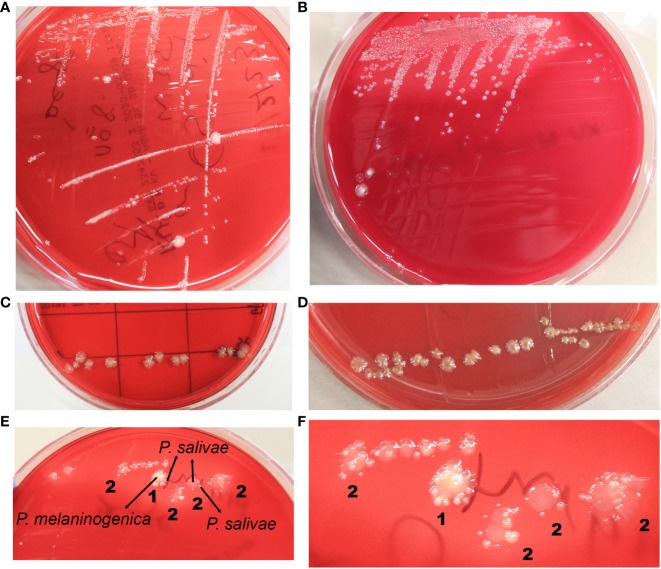
Diverse morphological and growth patterns of *Prevotella salivae.* **(A)** Illustrates a single strain of *P. salivae* (OMI1400), showcasing distinct sizes, shapes, and colors on the same agar medium. **(B)** Additional morphologies of *P. salivae* (OMI1399). **(C)** *P. salivae* (OMI1399) after one week of incubation. **(D)** *P. salivae* (OMI1399) after two weeks of incubation, with a noticeable central bull-eyed pigmentation. **(E)** 1. Satellite colonies of *P. salivae* (small peripheral) (OMI1400) orbiting a central colony of *P. melaninogenica* (OMI1328). 2. Self-satellitism of *P. salivae* (OMI1400), depicting small peripheral colonies encompassing a larger colony of itself or the identical species. **(F)** 1. Magnified section of Figure E (1) distinctly showcasing the satellitism interaction between *P. salivae* and *P. melaninogenica*. 2. An enlarged section of Figure E (2), explicitly illustrating the self-satellitism phenomenon of *P. salivae*.

Distinct colonies of *P. salivae* manifested varying colors and sizes. On Schaedler KV agar medium, three different sizes were discernible: large, medium, and small (**Figures 1A, B**). These colonies exhibited a spectrum of colors, ranging from brownish beige to beige to a greyish turbidity. Importantly, color darkening occurred after prolonged incubation periods (**Figures 1C, D**). Notably, large *P. salivae* colonies exhibited no fluorescence when exposed to UV light, rendering their isolation more intricate. However, smaller colonies exhibited a light brick-red fluorescence under UV light.

Throughout multiple isolation attempts of *P. salivae* from mixed cultures, remarkable observations swiftly became evident. Intriguingly, it was observed on the agar plate that *P. salivae* consistently coexisted in close proximity to *P. melaninogenica*, frequently displaying a pattern in which small peripheral *P. salivae* colonies encircling a central cluster of *P. melaninogenica*, a phenomenon we will refer to as “satellitism” (**Figures 1E, F**). Another noteworthy observation was the presence of a self-satellitism phenomenon among *P. salivae*. This was evident in the form of small *P. salivae* colonies surrounding a larger central mass of *P. salivae* (**Figures 1E, F**). To validate these findings, all these *P. melaninogenica* and *P. salivae* isolates underwent rigorous identification and confirmation through MALDI-TOF analysis. It was discernible that successful colony formation occurred only after reaching a critical mass of cells (**Figures 1E, F**).

### Interplay of growth and inhibition between *P. salivae* and *P. melaninogenica* in cross-streak zones

**Figure 2:**
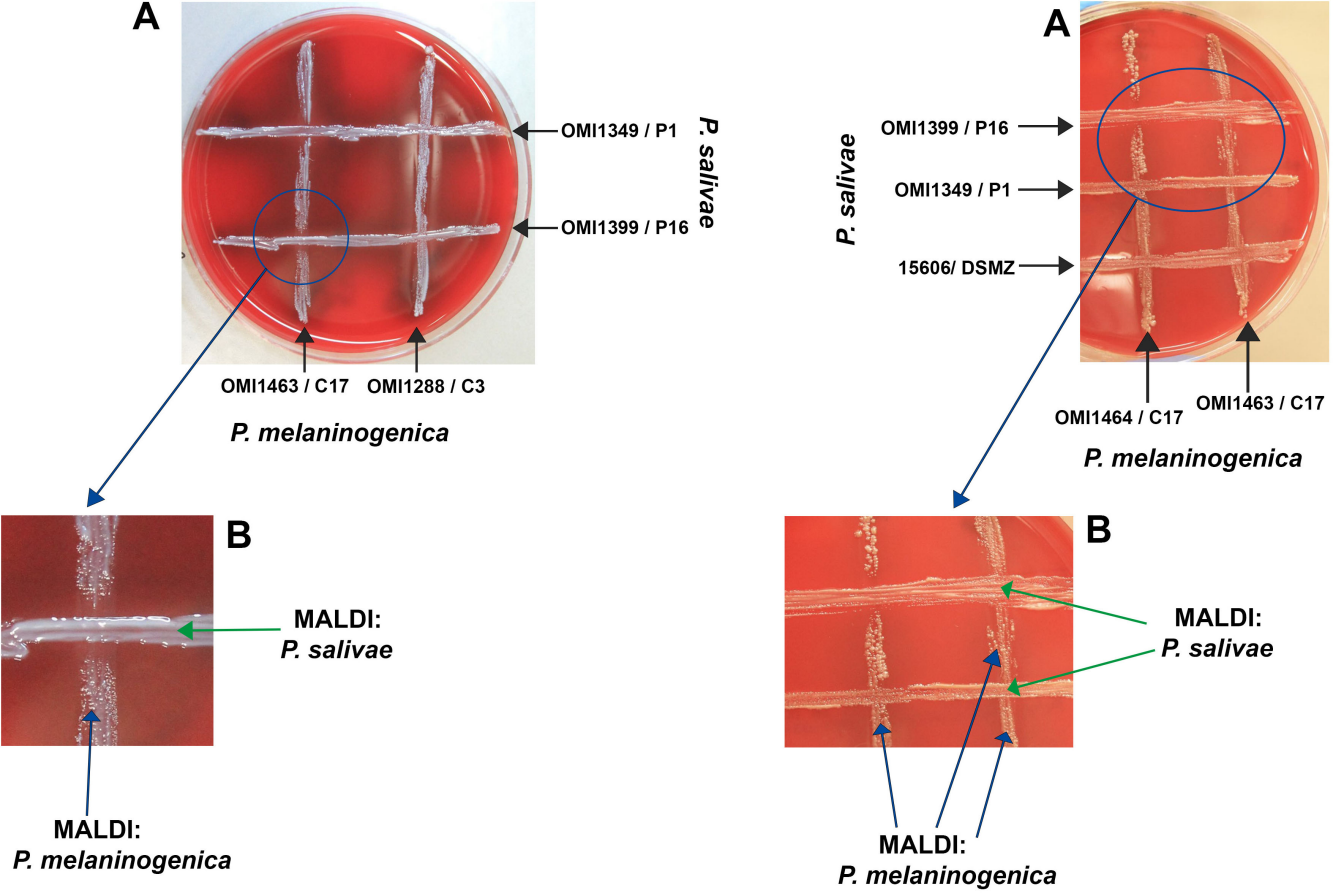
Growth and inhibition zones from *P. salivae* and *P. melaninogenica* cross-streaks. **(A)** Schaedler KV agar plate showing different *P. melaninogenica* strains (vertical) cross-streaked with different *P. salivae* strains (horizontal). **(B)** Enlarged intersection between *P. salivae* and *P. melaninogenica* streaks clearly showing the inhibition of *P. melaninogenica* in the contact zone with *P. salivae*.

This experiment aimed to elucidate the nature of the interaction—whether inhibitory or supportive—between *P. salivae* and *P. melaninogenica*, particularly given their proximity in certain instances. Emphasis was placed on monitoring the growth of both bacteria at the intersections of the cross-streaks. Initial findings revealed that, in the absence of *P. melaninogenica*, *P. salivae* exhibited limited growth, except for the reference strain DSMZ 15606. Interestingly, a significant expansion of *P. salivae* colonies was observed in the presence of *P. melaninogenica*, culminating in the subsequent inhibition of *P. melaninogenica* growth. It’s worth noting that these observed effects were contingent on factors such as bacterial strain, growth phase, and colony size. Consequently, the experiments were meticulously repeated to ensure the validity of these observations. In instances where uncertainties arose, the identity of colonies within the crossing area was confirmed through MALDI-TOF results (**Figures 2A, B**).

### Molecular typing using the taxonomic 16S rRNA gene

**Figure 3:**
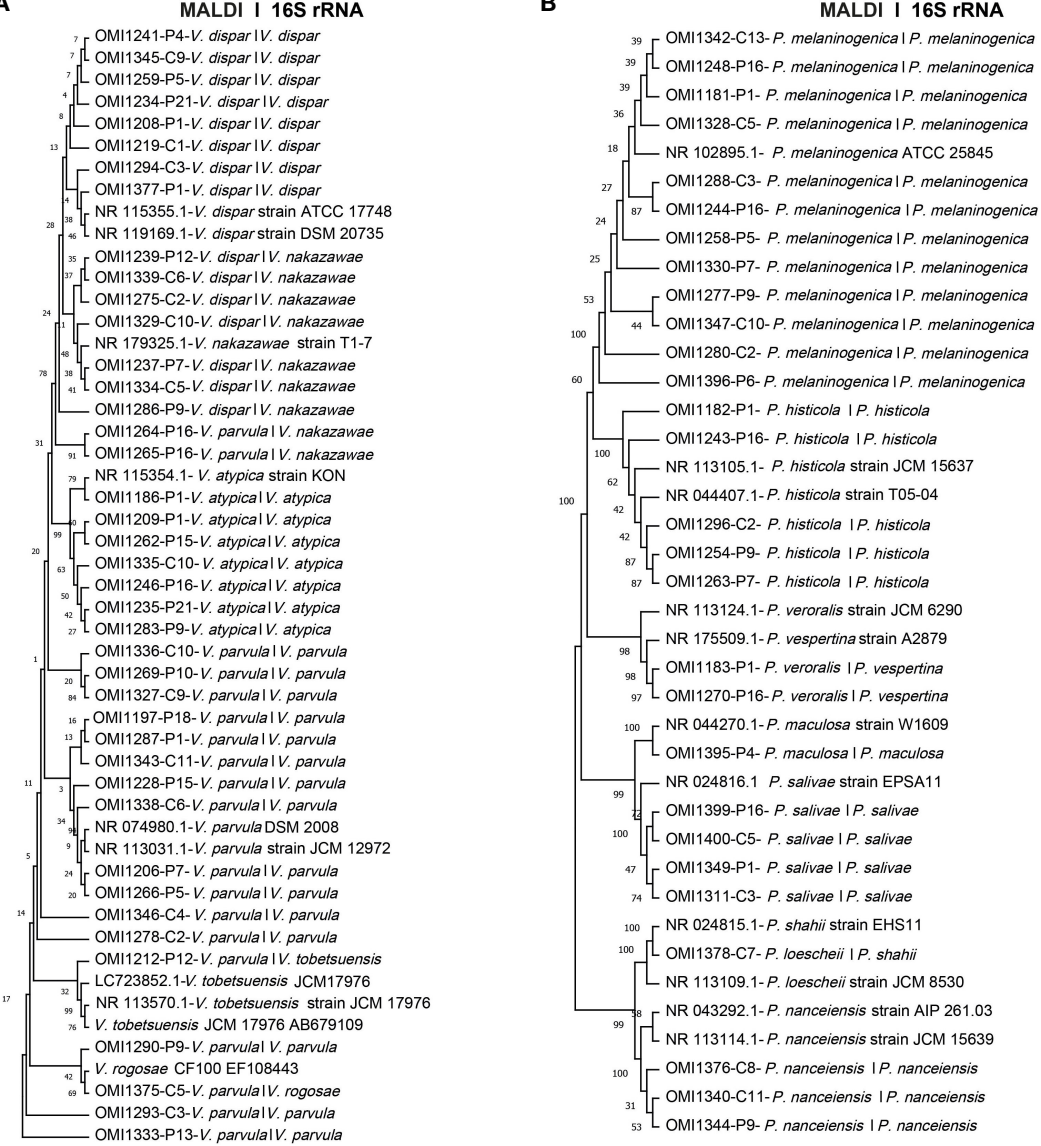
Unrooted phylogenetic trees based on 16S rRNA sequences, comparing MALDI-TOF and 16S rRNA sequencing identifications. The first name indicates species identification by MALDI-TOF, while the second name represents the identification based on 16S rRNA sequence analysis. **(A)** Phylogenetic tree of 41 *Veillonella* spp. isolates **(B)** Phylogenetic tree of the 28 investigated *Prevotella* spp. isolates.

Utilizing molecular-based techniques, we meticulously selected 41 *Veillonella* spp. and 28 *Prevotella* spp. isolates for comprehensive species identification. This approach involved the amplification and sequencing of the taxonomic 16S rRNA gene. These isolates were carefully chosen, representing recurrent species within each IBD patient and healthy control group, as previously outlined. Notably, 11 *Veillonella* spp. and 3 *Prevotella* spp. isolates exhibited species identification discrepancies between the MALDI-TOF and 16S rRNA sequencing. For example, strains such as OMI1237, OMI1286, OMI1239, OMI1275, OMI1334, OMI1339, and OMI1329 were identified by MALDI-TOF as *V. dispar* but as *V. nakazawae* via BLAST analysis. Similarly, while MALDI-TOF identified OMI1264 and OMI1265 as *V. parvula*, BLAST recognized them as *V. nakazawae*. Instances like OMI1212 were recognized by MALDI-TOF as *V. parvula* but as *V. tobetsuensis* by BLAST. Likewise, OMI1375 was classified by MALDI-TOF as *V. parvula* and as *V. rogosae* by BLAST (**Figure 3A**).

As for *Prevotella*, the strains OMI1183 and OMI1270, identified by MALDI-TOF as *P. veroralis*, were classified as *P. vespertina* according to BLAST. In addition, OMI1378, recognized as *P. loescheii* by MALDI-TOF, was identified as *P. shahii* by BLAST. Nevertheless, for the majority of *Veillonella* spp. (30 isolates) and *Prevotella* spp. (25 isolates), BLAST results concurred with MALDI-TOF identifications (**Figure 3B**). To our knowledge, this is the first study isolating *P. salivae* and *P. shahii* from the saliva of IBD patients.

### Quantification of *P. salivae* using RT-qPCR and comparison with 16S rRNA amplicon sequencing

The assessment of absolute abundance for the three target groups—*Veillonella* genus, *Prevotella* genus, and *P. salivae* species—was conducted through two distinct methodologies: i) RT-qPCR: This method employed specific PCR primers, as previously detailed. ii) Comparative analysis: The 16S rRNA amplicon sequencing outcomes from the earlier study (Abdelbary et al., 2022) served as a basis. The calculation of absolute abundance involved multiplying the relative abundance by the total bacterial count, as previously elucidated.

**Figure 4:**
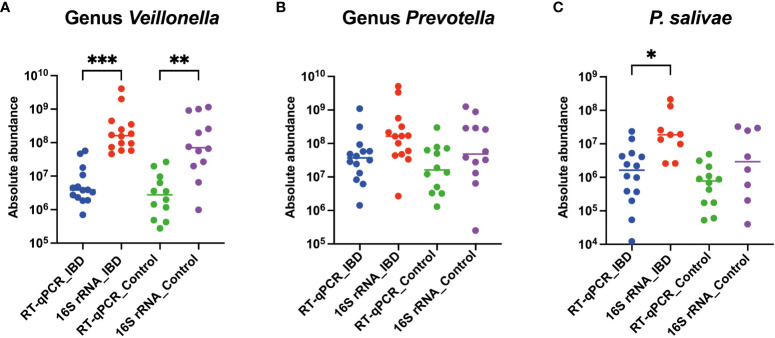
Comparative analysis of absolute abundance: RT-qPCR vs. 16S rRNA amplicon sequencing in both IBD patients and healthy controls for **(A)** *Veillonella* genus, **(B)** *Prevotella* genus, and **(C)** *P. salivae*. Significance levels are denoted as (*), (**), and (***) for *P* < 0.05, *P* < 0.01, and *P* < 0.001, respectively.

Interestingly, a recurring trend emerged, revealing that the absolute abundance calculated through 16S rRNA amplicon sequencing consistently exceeded that obtained via the RT-qPCR approach, both among patients and healthy controls. Specifically, the absolute abundance of the *Veillonella* genus in IBD patients (*P*<0.001) and controls (*P*<0.01) was significantly elevated when assessed using the 16S rRNA amplicon sequencing technique, compared to RT-qPCR (**Figure 4A**). Furthermore, the 16S rRNA amplicon sequencing method unveiled a more substantial absolute abundance of the *Prevotella* genus in both IBD patients and controls, in contrast to the RT-qPCR approach. However, this disparity did not attain statistical significance (**Figure 4B**). Finally, among IBD patients, the absolute abundance of *P. salivae*—determined through the 16S rRNA amplicon sequencing methodology—exhibited a considerable increase compared to RT-qPCR, while this distinction was not statistically notable when comparing healthy controls (**Figure 4C**). Taken together, our comprehensive analysis utilizing both the 16S rRNA amplicon sequencing and RT-qPCR methods consistently revealed heightened absolute abundance across all three bacterial groups examined in IBD patients, as compared to their counterparts in the healthy control group.

## Discussion

In this study, we conducted a comprehensive analysis of the prevalence and significance of *Veillonella* spp. and *Prevotella* spp. – with a focus on *P. salivae* – in IBD patients compared to healthy controls. Our approach encompassed diverse conventional cultivation methods, utilizing various agar media. Molecular species identification was accomplished through sequencing the taxonomic 16S rRNA gene (V1-V9) of isolated *Veillonella*, *Prevotella*, and *P. salivae* strains. Additionally, we employed RT-qPCRs to quantitatively assess the *Veillonella* spp., *Prevotella* spp., and *P. salivae* groups. To provide context, our findings will be interpreted in relation to the microbiome data from the same samples, as previously published (Abdelbary et al., 2022).

### High prevalence of *Veillonella* spp. and *Prevotella* spp. in IBD patients

Culture-based methods have been a mainstay in clinical microbiology for decades, although their labor-intensive nature poses challenges for comprehensive microbiome analysis. This is particularly true for anaerobic and low-abundance microorganisms, which are difficult to culture using conventional techniques (Damhorst et al., 2020). Nonetheless, tracking viable bacteria at the species level remains crucial. Thus, employing traditional cultivation methods, *Veillonella* spp., *Prevotella* spp., and *P. salivae* were successfully isolated from both IBD patients and healthy controls. Our results align with previous studies (Said et al., 2014; Z. Xu et al., 2018; Abdelbary et al., 2022; Balto et al., 2023), indicating a higher prevalence of *Veillonella* spp. and *Prevotella* spp. in IBD patients compared to healthy controls. However, it is noteworthy that *Veillonella* spp. was neither isolated from saliva samples of patients P3 and P6, nor was *Prevotella* spp. isolated from patients P10, P12, P13, and P21. This observation may be attributed to the decreased abundance of *Veillonella* spp. and *Prevotella* spp. in the remission phase of IBD patients, as previously reported (Zhang et al., 2020; Xia et al., 2022). Notably, the majority of patients in this study were in the remission phase.

The cultivation of *P. salivae* proved to be a challenging and labor-intensive process. Despite employing a variety of agar media enriched with diverse supplements and antimicrobial agents aimed at reducing gram-positive bacteria, our efforts persisted. Notably, GC-Lect agar, chosen for its known capacity to facilitate the growth of fastidious bacteria, was also incorporated into the study. Despite these measures, successful isolation of *P. salivae* was achieved from just two IBD patients and three control samples. Notably, one of the control samples was freshly collected saliva, which was included as an additional control. The immediate isolation of *P. salivae* from the fresh sample may imply the bacterium’s particular fastidious nature, necessitating specific environmental factors, nutrients, and potentially interactions with other species to flourish.

The presence of multiple other bacterial species on the agar plates posed a hindrance to *P. salivae*’s growth. Faced with intense competition for essential nutrients, *P. salivae* struggled to thrive, thus illustrating the principles of Darwin’s “survival of the fittest.” This intricate competition for resources rendered the isolation of *P. salivae* from undiluted saliva samples sub-cultured on agar plates remarkably challenging. Intriguingly, the dilution process appeared to facilitate the isolation of *P. salivae* by reducing the number of colonies on the agar plate, thereby easing the competition for nutrients (Sakamoto et al., 2000).

The literature on the cultivation and isolation of *P. salivae* has remained sparse, primarily characterized by the predominant application of molecular techniques in related studies. The original description by Sakamoto et al. is based on a single isolate, detailing colonies measuring 1–2 mm in diameter, characterized by shades of grey to light brown. These colonies exhibited circular, intact, slightly convex, and smooth features (Sakamoto et al., 2004). However, this description offers limited insight into potential variations among strains and different agar types. Additionally, the bacterium’s tolerance to oxygen remains largely unknown. It is intriguing to note that research has revealed that agar by-products released during autoclaving can interact with phosphate in the culture medium, resulting in the generation of hydrogen peroxide. This compound, in turn, impedes the growth of anaerobic species such as *Prevotella* (Tanaka et al., 2014; Kawasaki and Kamagata, 2017).

Notably, our study introduces a novel finding: evidence of self-satellitism in *P. salivae*. This phenomenon is characterized by the presence of smaller peripheral colonies forming a ring around a larger, mature central “mother colony.” The significance of an ample mass of the mother colony in facilitating effective satellite formation is highlighted—an observation previously documented only in the case of *Prevotella* sp. HOT-376 (Vartoukian, Adamowska, et al., 2016a). These novel findings underscore the need for further investigation to assess and optimize the cultivation techniques of *P. salivae* from the complex salivary microbiome.

The technique of cross-streaking an agar plate with a helper bacterium has been previously employed to successfully isolate *Haemophilus* (utilizing a *Staphylococcus aureus* helper-strain) or to cultivate various fastidious oral bacteria, as indicated by previous studies (Vartoukian et al., 2010; Vartoukian, Adamowska, et al., 2016a; Vartoukian, Moazzez, et al., 2016b). An illustrative instance involves the utilization of *Fusobacterium nucleatum* culture filtrate, which facilitated the growth of *Prevotella* sp. HOT-376. Additionally, it has been demonstrated that *Anaerolineae* HOT-439, *Bacteroidetes* HOT-365 and *Bacteroidaceae* HOT-272 exhibit a reliance on one or both of the helper bacteria *Cutibacterium acnes* and *F. nucleatum*. Notably, *C. acnes* and *P. intermedia* played crucial roles as helpers for promoting the growth of *Tannerella* sp. HOT-286 (Vartoukian, Adamowska, et al., 2016a; Vartoukian, Moazzez, et al., 2016b). Based on our relatively limited experience, it is evident that *P. salivae* is a bacterium with a delicate constitution, exhibiting intolerance to oxygen, and necessitating a span of five to seven days for its optimal growth on agar plates. Intriguingly, in our investigations, *P. salivae* was frequently observed in close adjacency to *P. melaninogenica* colonies, exhibiting a phenomenon akin to the satellite concept, wherein *P. salivae* seemed to orbit *P. melaninogenica*. This observation hints at a potential dependence of *P. salivae* on the presence of *P. melaninogenica*, as visually represented in **Figures 1E, F**. The results of cross-streaking experiments also unveiled that *P. melaninogenica* might act as a nurturing influence for *P. salivae*, fostering its growth until it reaches a certain colony size and/or quantity. An unprecedented observation that emerged from these experiments was the phenomenon where, after achieving a certain level of growth, *P. salivae* appeared to hinder the growth of *P. melaninogenica* (**Figure 2**). It is worth noting that the outcome of cross-streaking experiments was influenced by factors such as strain type, growth stage, and cell quantity. To unravel the precise relationship between these two *Prevotella* species, it is highly advisable to conduct further investigations using a more extensive strain collection.

### Enhanced efficiency of identifying *Veillonella* spp. and *Prevotella* spp. using 16S rRNA gene molecular typing compared to MALDI-TOF

In clinical laboratories, microorganisms are increasingly being identified using MALDI-TOF MS, a technique that records protein mass spectra from whole cell or intracellular content and compares them to known database references (Fenselau and Demirev, 2001; Lay, 2001). Accurate identification requires high spectrum quality and close/multiple matches to database references. Clinically significant species are commonly identified using popular commercial platforms such as Biotyper (Bruker Daltonics) and Vitek MS (BioMérieux). However, these methods often fall short when applied to environmental or newly described isolates, as reference databases only partially cover the vast diversity of microbial life (Strejcek et al., 2018). Therefore, reference-based MALDI-TOF MS classification has proven effective for identifying bacteria at the genus level, yet the limited references in databases restrict its accuracy at the species level (Strejcek et al., 2018). This limitation arises from missing reference strains in the MALDI-TOF Bruker database or underrepresentation of certain species, some of which are only represented by a few or even a single strain. For instance, in our study, *V. nakazawae* and *P. vespertina* were absent in the Bruker database but present in NCBI BLAST, leading to discrepancies.

Both MALDI-TOF and traditional bacterial identification methods, which are based on phenotypic traits, are generally less precise than identification through genotypic approaches (Clarridge, 2004). Analysis of the sequence similarity of the 16S rRNA gene remains a gold standard for bacterial species identification (Conville et al., 2017). Through an analysis of 6,787 genomes, a sequence similarity threshold of 98.65% was determined as optimal for delineating bacterial species (Kim et al., 2014). Even the identification of non-cultured bacteria, poorly documented species, infrequently isolated strains, or atypical phenotypes is possible using 16S rRNA gene sequence analysis (Clarridge, 2004). Consistent with previous studies (Clarridge, 2004; Strejcek et al., 2018), our investigation reveals that MALDI-TOF and 16S rRNA classification did not consistently align (see **Figure 3**). These findings align with earlier research indicating that the agreement between MALDI BioTyper and 16S rRNA gene analysis ranged from 41% to 92.2% across samples (Mellmann et al., 2008; Bizzini et al., 2011; Schmitt et al., 2013; Cheng et al., 2015; Fykse et al., 2015; Schulthess et al., 2016). The lack of a critical number of well-referenced strains in the databases likely contributes to these discrepancies.

### Impact of method and primer coverage on absolute abundance estimation

Addressing the challenges posed by culture-based approaches, RT-qPCR has emerged as a valuable tool for enhancing diagnostic sensitivity and lowering the limit of detection, particularly for fastidious organisms (Damhorst et al., 2020). This heightened sensitivity is evident in the considerably higher absolute counts of *P. salivae* observed in both IBD patients and controls when assessed through RT-qPCR, compared to the number of colonies/strains isolated via cultivation. Notably, the quantification of *Prevotella* spp. in general exhibited substantial discrepancies in P10, P12, P13, and P21 samples, reflecting the intrinsic challenges associated with cultivating and isolating *Prevotella* spp. from these particular saliva samples. However, it’s important to acknowledge that quantifying bacterial load via RT-qPCR can lead to divergent profiles (Galazzo et al., 2020).

The application of RT-qPCR does come with certain limitations. It necessitates the pre-specification of amplification targets, and the selection of an external reference organism introduces a potential bias. To mitigate this, various common microbiological taxa can serve as reference organisms for the standard curve (Bonk et al., 2018). Nonetheless, the choice of reference organisms may introduce variations in quantification findings due to differences in qPCR amplification efficiency among reference species. This poses a critical question regarding what best represents a microbiome – relative or absolute numbers of bacterial taxa. While relative abundance provides an overall proportional view, it might not accurately capture significant shifts in microbiota where changes in absolute quantities are pivotal (Gloor et al., 2017). An approach based on estimated absolute abundance, as seen in this study, offers a distinct and more realistic perspective (Azarbad et al., 2022).

Prior research has illuminated the impact of primer coverage on accurate microbial diversity assessment (Mao et al., 2012; Conrads and Abdelbary, 2019; Zhang et al., 2020). Primer coverage is a critical factor, potentially leading to underestimation or overestimation of microbial diversity. Our study reveals that the lower absolute abundance observed with the RT-qPCR method, in comparison to 16S rRNA amplicon sequencing data, could be attributed to the lower coverage/higher specificity of RT-qPCR primers. For instance, the primer coverage for *Veillonella* species was 72% with RT-qPCR, as opposed to 81.5% with 16S rRNA amplicon sequencing. Similarly, for *Prevotella* species, the RT-qPCR primer coverage was 68.5%, while the 16S rRNA amplicon sequencing primer coverage was 90.4%. This divergence is also evident in the case of *P. salivae*, where the broader coverage of the 16S rRNA amplicon sequencing primer contributes to a greater absolute abundance measurement (**Table 1**). Furthermore, the choice of a single reference bacterial species may have introduced variations in quantification findings when comparing results obtained through the RT-qPCR method with those derived from 16S rRNA amplicon sequencing. To mitigate the potential for considerable inaccuracies, it has been previously recommended to always employ at least two reference genes (Nicot et al., 2005). However, future research utilizing the digital polymerase chain reaction (dPCR) approach holds promise for resolving discrepancies stemming from the use of external reference organisms, as dPCR eliminates the need for such references. Beyond quantifying absolute copy numbers, dPCR outperforms qPCR by detecting even minor fold-change variations. It produces results that are not only more accurate than using qPCR, but also more reproducible and statistically significant, meeting the rigorous standards required for high-quality data (Taylor et al., 2017).

In conclusion, absolute quantification data is heavily influenced by primer choice, necessitating caution when comparing abundance data across studies unless identical primer sets are employed (Wintzingerode et al., 1997; Orschler et al., 2019; Jian et al., 2020). Moreover, the selection of primers from different hypervariable regions of the 16S rRNA gene can yield substantially different results, with primers from the same region providing more comparable outcomes (Engelbrektson et al., 2010; Orschler et al., 2019). For absolute abundance calculations, our reliance on RT-qPCR targeting the V3-V4 hypervariable regions, similar to a previous study (Abdelbary et al., 2022), was still associated with lower coverage compared to 16S rRNA amplicon sequencing. This discrepancy can be attributed to the ambiguities present in the 16S rRNA amplicon sequencing primers, which hinder their use in RT-qPCR. Despite these complexities, this investigation underscores the importance of considering methodological nuances in accurate microbial quantification.

While this study provides valuable insights, few limitations need to be acknowledged. The relatively small sample size restricts definitive conclusions regarding significance. Furthermore, factors impacting the microbiome, such as diet, smoking, oral inflammation, and oral hygiene, were not controlled for prior to sample collection, even though they are important parameters (Signoretto et al., 2006; Mason et al., 2013; X. Xu et al., 2015).

Despite these limitations, our findings highlight the higher prevalence of *Veillonella* spp., *Prevotella* spp., and *P. salivae* in IBD patients compared to healthy controls. Additionally, the study sheds light on the optimal cultivation conditions for *P. salivae* and the potential role of helper strains in its growth. The implications of helper-dependency, whether self or reliant on *P. melaninogenica*, warrant further exploration to unravel the clinical significance of *P. salivae*. Ultimately, our findings highlight the significance of primer coverage, as demonstrated by the broader scope of the 16S rRNA amplicon sequencing primer, leading to a more comprehensive assessment of absolute abundance.

## Author contributions

MH: Data curation, Investigation, Visualization, Writing – original draft, Writing – review and editing. GC: Writing – review and editing, Funding acquisition. MA: Funding acquisition, Writing – review and editing, Conceptualization, Data curation, Investigation, Methodology, Project administration, Software, Supervision, Validation, Visualization.

## Supplementary material

Supplementary Table 1 MALDI-TOF identity and number of *Veillonella* spp. isolates in IBD patients and healthy controls using different agar media.

Supplementary Table 2 MALDI-TOF identity and number of *Prevotella* spp. isolates in IBD patients and healthy controls using different agar media.

Supplementary Table 3 All reference strains used in phylogenetic trees along with their accession numbers.
